# Improving Cross‐Cultural Dementia Screening: Evaluating A Pilot Training for Community Health Staff

**DOI:** 10.1002/alz70858_096747

**Published:** 2025-12-24

**Authors:** Freddie O'Donald, Clara Calia

**Affiliations:** ^1^ NHS Tayside, Dundee, United Kingdom; ^2^ School of Health in Social Science, University of Edinburgh, Edinburgh, United Kingdom

## Abstract

**Background:**

Cross‐cultural assessment of dementia refers to evaluating cognitive and functional decline while accounting for cultural, linguistic, and socioeconomic factors that may influence the presentation of symptoms and performance on assessment tools. This approach is essential to avoid misdiagnosis and ensure equitable care for individuals from diverse backgrounds. However, many healthcare professionals may lack the skills and knowledge required for culturally sensitive dementia assessments. This pilot training session evaluated the effectiveness of a brief workshop designed to improve community healthcare staff's ability to perform cross‐cultural dementia assessments.

**Methods:**

The pilot program consisted of a three‐hour interactive workshop for community healthcare staff working with older adults in an NHS mental health service in Berkshire. The workshop included education on how culture can impact dementia presentations, the use of validated culturally appropriate assessment tools, and strategies for overcoming linguistic barriers within assessments. A pre‐ and post‐workshop survey (using a ten‐point Likert scale) measured participants’ knowledge, confidence, and awareness of cross‐cultural assessment tools. Open‐ended questions captured qualitative feedback on the training content and its relevance to clinical practice.

**Results:**

Seventeen staff members participated in the training, and their knowledge, confidence, and awareness significantly improved afterwards. Mean knowledge scores increased from 5.2 (SD = 1.4) pre‐training to 8.7 (SD = 0.8) post‐training (*p* < 0.001). Confidence ratings rose from 2.8 (SD = 0.9) to 6.4 (SD = 0.6) (*p* < 0.001). Awareness of culturally appropriate assessment tools also improved significantly, increasing from 2.3 (SD = 1.2) pre‐training to 8.9 (SD = 0.7) post‐training (*p* < 0.001). Qualitative feedback suggested that participants found the training helpful, with case discussions particularly impactful. Several participants expressed a need for further opportunities to practise applying these skills in real‐world settings.

**Conclusions:**

This pilot training program effectively improved the knowledge, confidence, and awareness of community older adult mental health staff in conducting cross‐cultural dementia assessments, highlighting its potential to enhance diagnostic accuracy and equity in diverse populations. Future research should investigate the long‐term impact of such training on clinical practices and patient outcomes and explore how to integrate ongoing support and hands‐on application into training programs.

